# Mechanism of PKM2 affecting cancer immunity and metabolism in Tumor Microenvironment

**DOI:** 10.7150/jca.54430

**Published:** 2021-04-24

**Authors:** Mengxi Chen, Huan Liu, Zhang Li, Alex Lau Ming, Honglei Chen

**Affiliations:** 1 Department of Pathology, Zhongnan Hospital of Wuhan University, Wuhan 430071, P. R. China.; 2 Department of Pathology, School of Basic Medical Sciences, Wuhan University, Wuhan 430071, P. R. China.

**Keywords:** PKM-2, PD-L1, cancer immunity, metabolism

## Abstract

PKM2 is the enzyme that regulates the final rate-limiting step of glycolysis. PKM2 expression can reinforce the utilization of oxygen and synthesis of growth substances in cancer cells by enhancing OXPHOS and the Warburg effect. In cancer immunity, PKM2 can modulate the expression of PD-L1 in M2 macrophage and decrease the amount and activity of CD8^+^ T cells. This affects cancer cell killing and immune escape sequentially. How PKM2 regulates PD-L1 expression through immunometabolism is summarized. PKM2 builds a bridge between energy metabolism and cancer immunity. The activator and inhibitor of PKM2 both promote the anti-cancer immune response and inhibit cancer growth and metastasis by regulating the metabolism of cancer cells and immune cells in the tumor microenvironment through HIF-1α/PKM2 pathway. This review focuses on the precise role of PKM2 modulating immunometabolism, providing valuable suggestions for further study in this field.

## Introduction

Pyruvate kinase is an enzyme that catalyzes the conversion of phosphoenolpyruvate (PEP) and ADP to pyruvate and ATP in glycolysis, and plays a role in regulating cell metabolism 1, 2. There are four pyruvate kinase isoforms (PKM1, PKM2, PKR, PKL), which can express in the different organizations and play different roles 3. PKL is mainly distributed in liver, kidney and red blood cells. PKM1 is mostly found in myocardium, skeletal muscle and brain tissue. PKM2 can be expressed in normal cells of lung, fat cells, retina, islets and employed in nucleic acid synthesis, and also expressed in proliferating cells, such as embryonic stem cells, cancer cells, and immune cells 4. In normal tissue differentiation, PKM2 will gradually disappear. While in caner formation, PKM1 or PKML/R gradually disappears, and PKM2 is markedly upregulated, ultimately becoming a cancer-specific pyruvate kinase 5. Previous work demonstrated that PKM2, a preferred splice isoform of pyruvate kinase in cancer cells that converts PEP to pyruvate as the final step of glycolysis, is critical for aerobic glycolysis in cancer cells 6. PKM2 is distinct in that, it can exist in two functional forms: the active tetrameric form and the inactive dimeric form 7. Dimeric PKM2 has a low affinity for PEP, while tetrameric PKM2 has a higher affinity for PEP. The low affinity of dimeric PKM2 for PEP results in a low glycolytic rate, which in turn leads a high accumulation of glycolytic intermediates 8. During carcinogenesis, PKM2, an enzyme involved in the last and rate-limiting step of glycolysis, is converted to its dimeric form within cancer cells 9, which increases glucose uptake and facilitates the accumulation of glycolytic intermediates important for anabolic processes such as the synthesis of nucleic acids, amino acids, and lipids 6, 10, 11. PKM2 is highly expressed in aggressive cancer cells compared to normal cells 12. Patients with high PKM2 expression have reduced overall and disease-free survival of lung adenocarcinoma as compared to those with low PKM2 expression 13. Knockdown of PKM2 significantly inhibits the growth of cancer cells 14. Thus, these metabolic differences provide a highly valuable potential target for the development of new drugs. TEPP-46 and DASA-58, which are PKM2 activators, can bind PKM2 through a binding pocket distinct from that of fructose-1,6-bisphosphatase (FBP) and that they stabilize a tetrameric conformational state, which may impair cancer cell proliferation by interfering with anabolic metabolism 15.

Recent studies have shown that PKM2 is an important factor in the Warburg effect of cancer cells, immune cells (such as lymphocytes, dendritic cells, macrophages) and immune checkpoints [such as programmed cell death ligand-1 (PD-L1)] in the cancer immune microenvironment, and PKM-2 has different functions in the occurrence, progression and metastasis of cancer 16-18. However, whether PKM2 plays a vital role in cancer metabolism and immunity is unclear. Exploring the correlation between the two is crucial for the study of PKM2 as a potential target for cancer therapy. Based on current research, this review mainly focuses on the precise role of PKM2 in the development of cancer and immunometabolism, and integrates the conclusions of recent studies to further analyze the molecular mechanism of cancer growth that PKM2 is a part of.

## Structure and functions of PKM2

### Structure of PKM2

PKM1 and PKM2 are encoded by the same gene, and differ by alternative splicing of exons 9 and 10. Human PKM1 is different from PKM2 only in 23 amino acids, and these 23 amino acids are concentrated in 56 amino acid fragments, which is why PKM1 and PKM2 differs in their kinetic properties and regulatory mechanisms. Human PKM2 is composed of N region, A1 region, A2 region and C region, with a total of 513 amino acids 19. Among them, zone A is responsible for coupling monomers into dimers, and zone C is responsible for coupling two dimers into tetramers. PKM2 is available in both dimeric and tetrameric forms, but other subtypes of pyruvate kinase are tetramers.

### Functions of PKM2

The PKM2 dimer cause the glucose produce lactic acid or pyruvate through glycolysis, which is used to synthesize nucleotides, amino acids, phospholipids and other macromolecular biological substances through the pentose phosphate pathway. Phospholipids are a raw material for tumor growth and an important structure of cancer cells. PKM2 tetramer can degrade glucose to pyruvate and lactic acid and produce energy. The conversion of PKM2 in these two forms enhances the ability of cancer cells to compete with normal cells for survival and growth in different environments 7. The dimer form of PKM2 is dominant in cancer cells, so the dimeric PKM2 is also known as cancerous M2-PK. Dimeric PKM2 is released from the tumor tissue into the patient's blood and feces, so quantitative detection of PKM2 in plasma and feces may be used as an early diagnostic method for tumors. On the other hand, activation of PKM2 will convert the dimer form to the tetramer form, reducing the synthesis of substances needed for cancer cell growth 20. PKM2 is activated by activators such as FBP and TEPP-46, may be a potential target for lung cancer treatment *in vitro* and* in vivo* 21, 22. Therefore, further study of the prevalence of PKM2 in the cancer microenvironment, and its roles in the carcinogenesis are of great significance.

## Regulation of PKM2

The enzymatic activity of PKM2 is related to the stereo conformation of PKM2, and the changes in spatial structure of PKM2 is allosterically regulated by endogenous and exogenous activators and inhibitors. PKM2 has PK enzyme activity when it serves as a tetramer, and dimeric PKM2 regulates the step of glycolysis that shifts the glucose metabolism form the normal respiratory chain to lactate production in cancer cells, so dimeric PKM2 can decrease pyruvate kinase activity 23. PKM2 is activated by the glycolytic intermediate products named FBP. It can also be activated by the allosteric effects of serine and succinylaminoimidazolecarboxamide ribose-50 phosphate SDH succinate dehydrogenase (SAICAR) 24, 25. The PK enzyme activity of PKM2 can also be inhibited by many endogenous inhibitors and cell signaling events including 0-GlcNAcylation, pyruvate (PYR), p-tyrosine (P-TYR), phenylalanine (PHE), alanine (ALA), adenosine triphosphate (ATP), and thyroid hormone T3^26^-^28^. In addition, PKM2 post-translational modification (PTM) also involves many molecules. P300 can acetylate PKM2 at the K433 site, and PKM2 K433 acetylation can convert cell proliferation and cytoplasmic metabolism kinase into nuclear protein kinase activity 29. Parkin promotes the ubiquitination of Lys186 and Lys206 in PKM2, thereby inhibiting the biological activity of PKM2 and regulating glucose metabolism 30. Proviral insertion in murine lymphomas 2 (PIM2) can phosphorylate the Thr454 site of PKM2, mediate PKM2-dependent anaerobic glycolysis, and maintain the mitochondrial function of cancer cells 31. Prolyl hydroxylase 3 (PHD3) can hydroxylate the Pro403 and Pro408 sites of PKM2, when PKM2 binds to PDH3. The modified PKM2 is easier to freely bind to HIF-1 and forms an activation loop to promote anaerobic glycolysis and metabolic reorganization 32. MiR-4417 targets TRIM35 and regulates the phosphorylation of PKM2 Y105 to promote cell proliferation and inhibit cell apoptosis, and PKM2 Y105 phosphorylation promotes hepatocellular carcinoma growth 33.

It is now recognized that when some allosteric modulators participate in the allosteric regulation of the spatial structure of PKM2, the tetramer PKM2 can be transferred from the compact state (R state) to the loose state (T state), and finally decomposed into the dimer form 34. When these allosteric modulators bind to PKM2, they will change the spatial conformation of PKM2, influence the electrostatic force inside the molecule, and then affect the transition state of PKM2. PKM2 R state can form a tetramer and perform PK enzyme activity. After allosteric regulation, PKM2 forms a loose and unstable T state, and finally breaks the connecting fragments in the tetramer to form a PKM2 dimer form with lower PK enzyme activity. Although PK enzyme activity is low, it has protein activation ability 11.

## PKM2 regulates energy metabolism of cancer cells

### Warburg effect

Tumor characteristics include not only the loss in control of cell proliferation, but also the regulation of energy metabolism to supply the energy and metabolic intermediates needed for cell growth and division. The imbalance of cellular energy metabolism mostly reflects in the reprogramming of cellular energy metabolism. The normal cells of the body are mainly powered by high-efficiency aerobic oxidative phosphorylation (OXPHOS), and only undergo glycolysis under hypoxic conditions. However, the growth of cancer cells requires a large amount of energy and synthetic biomacromolecules (nucleotides, amino acids, lipids) in a short period. Both of which cannot be satisfied, as the oxidative phosphorylated tricarboxylic acid cycle takes a long time and the final products of metabolism are carbon dioxide and water. Therefore, cancer cells tend to undergo glycolysis under aerobic conditions, simultaneously, it is possible to synthesize pyruvate and lactic acid to carry out the pentose phosphate pathway to synthesize biomacromolecules to meet the needs of cancer cells. This is the famous Warburg effect 35, which makes cancer cells more competitive than normal cells under local hypoxia, and is also one of the main markers of cancer 36.

### PKM2 and Warburg effect

An important feature of energy metabolism in cancer cells is the enhancement of glycolytic enzyme activity and the transformation of glycolysis isoenzymes 37. PKM2 is present in the cytoplasm as a tetramer with high pyruvate activity, promoting ATP production. It exists as a dimer with high protein kinase activity in the nucleus, promoting the synthesis of macromolecular substances required for tumor growth 1. Conformational transformation of PKM2 is regulated by both intracellular and extracellular factors such as intermediate metabolites and protein post-translational modifications. Cancer cells require both energy production and biosynthesis, which can change the activity of PKM2 to meet these two needs through different metabolic pathways. Cancer growth and development are characteristically associated with synergistic cell proliferation and cell survival under stress. PKM2 contributes to these two effects 38-40. These promoting effects of PKM2 on cancers act through the Warburg effect, which allows cancer cells to undergo glycolysis in an aerobic environment. Intermediates products of glycolysis enter other secondary pathways such as the hexosamine pathway, uridine diphosphate glucose synthesis and glycerol synthesis. Experiments have also demonstrated that siRNA-mediated knockdown of PKM2 can significantly inhibit proliferation, glucose uptake (25%), ATP production (20%) and fatty acid synthesis in A549 cells, while mitochondrial respiration capacity of cells increased (13%), and suppress the expression of glucose transporter (GLUT1) and ATP citrate lyase, which is essential for fatty acid synthesis 41. Moreover, gene silencing of PKM2 can also repress the expression of matrix metalloproteinase 2 (MMP-2) and vascular endothelial growth factor (VEGF), which play important roles in the degradation of extracellular matrix and angiogenesis, respectively 41. Therefore, PKM2 can simultaneously activate the Warburg effect and lipid synthesis, thereby promoting the proliferation and invasion of cancer cells, and producing more NADPH to meet the needs of increasing cancer metabolism.

### PKM2 and OXPHOS

Since tumors still retain mitochondrial function, OXPHOS in mitochondria persist with PKM2 being closely related to mitochondrial biological functions of various cells. Mitochondrial dynamics affect OXPHOS 42-44. Mitochondria are highly dynamic organelles that continuously fuse the outer membrane and intimal fission to produce tubular or mitochondrial fragmentation 45, 46. Mitochondrial fusion is regulated by the GTPases dynamin family, such as mitofusin 1 (MFN1) and mitofusin 2 (MFN2), which regulates mitochondrial outer membrane fusion, and fusion promotes complementarity between damaged mitochondria 45, 47-49. Moreover, fission promotes mitochondrial adaptation through mitochondrial autophagy in a manner that is dependent on or independent of Parkin to promote mitochondrial damage 50-52.

PKM2 interacts with the key regulatory factor mitofusin, promotes mitochondrial fusion and oxidation, and attenuates glycolysis. mTOR can increase the interaction between PKM2 and MFN2 through MFN2 phosphorylation, thereby regulating the effects of PKM2 and MFN2 on glycolysis, mitochondrial fusion and OXPHOS 53. Thus, mTOR-MFN2-PKM2 signal axis regulates the growth of cancer cells by glycolysis and OXPHOS.

## PKM2 regulates cancer immunity

PKM2 plays a very important role in the occurrence, growth and metastasis of cancers. These processes are closely correlated to the internal and external environment in which cancer cells are located. This is the tumor microenvironment (TME). TME not only includes the structure, function and metabolism of the tissue, but it is also related to the intrinsic environment of the cancer cells themselves. TME is composed of blood vessels, myeloid-derived suppressor cells (MDSC), antigen-presenting cells (APC), lymphocytes, dendritic cells (DCs), fibroblasts, extracellular matrices, soluble factors such as cytokines and growth factors 54. These components play various roles in the anti-cancer immune response and have a vital impact on the development of cancers.

Nevertheless, energy metabolism is an important orchestrator of immune functions. Aerobic glycolysis has direct roles in controlling the differentiation and function of lymphocytes including T cells, B cells and Natural Killer cells. Glycolytic enzymes can control lymphocyte function through binding to mRNA and regulating protein synthesis 55. Thus, PKM2 has great significance to the energy metabolism and signal transduction of various immune cells and molecules in the TME, which provides a bridge between immunity and energy metabolism of cancers.

### PKM2 and T cells

#### PKM2 directly interacts T cells

When lymphocytes are activated, aerobic glycolysis is involved, which is manifested by an increase in glycolytic enzyme expression before lymphocyte proliferation 55, 56. After TCR (T cell receptor) stimulation, T cells up-regulate the expression of PKM2 and accumulate in the nucleus of CD4^+^ T cells 57. T cells preferentially express PKM2 rather than PKM1 in both resting and activated states, but mouse and human CD4^+^ T cells also upregulate the expression of PKM1 subtype after activation, and PKM1 is upregulated when CD3/CD28 is activated. These results are somewhat surprising, negative correlation has been observed between the expression of PKM1 and PKM2 in immune cells 58.

Dimeric PKM2 has also been shown to modulate the activity of mTORC1 in transformed cells, via phosphorylation of the mTORC1 inhibitor proline-rich AKT1 substrate 1 (AKT1S1) 59 or by reducing serine synthesis, which has been shown to sustain mTORC1 function in proliferating cells 60. At the same time, PKM2 can also control the functionality of HIF-1α, mTORC1, and myc and the engagement of aerobic glycolysis in TCR-activated CD4^+^ T cells, which are crucial determinants for their activation and effector functions 61. Furthermore, a study has shown that PKM2 controls T cell activation induced by homocysteine 62. Both TEPP-46 and DASA-58 can suppress the development of IL-17-producing T helper cell 17 (Th17) 63. Moreover, PKM2 tetramerization can specifically modulate the expression of certain Th17 and T helper cell 1 (Th1) specific transcription factors (Rora, Irf4, Runx1, and Eomes). Above all, PKM2 can regulate HIF-1α and mTOR activity, which can affect the differentiation of helper T cells 64.

**Figure 1:**
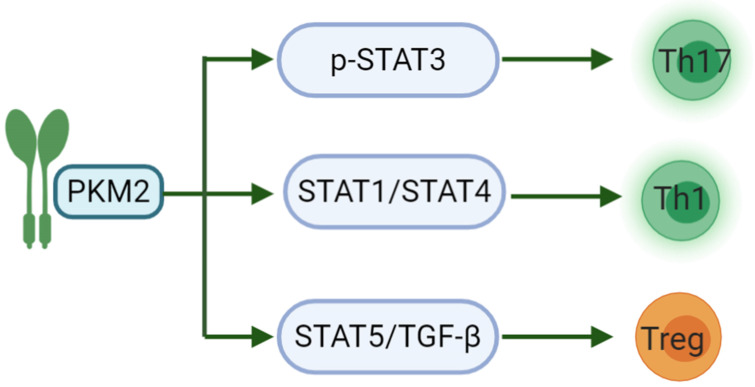
PKM2 acts on the STAT family in the differentiation of pro-inflammatory T cell subsets. PKM2 can regulate the phosphorylation of STAT3 to induce the differentiation of T cells into Th17 to participate in inflammation-related diseases; and then regulate STAT1/STAT4 to induce Th1 formation and partake in cellular immunity. The last, PKM2 can modulate STAT5 to combine with TGF-β, induce Treg formation and maintain immune tolerance.

Another study has shown that PKM2 may regulate the generation of different T cell subsets by affecting signal transduction and activator of transcription (STAT) proteins. STATs are important participants in the differentiation of pro-inflammatory T cell subsets, of which STAT3 affects the development of Th17. STAT1/STAT4 controls the production of Th1 and STAT5 regulates TGF-β is essential for Treg induction 65. IL-23 is an important cytokine for Th17 polarization and can cause phosphorylation-related nuclear translocation of PKM2 in T cells 66. However, dimeric PKM2 has been previously reported to translocate to the nucleus of cancer cells, where it could directly phosphorylate STAT3, inducing the expression of STAT3-dependent genes 67. Moreover, a recent study linked PKM2 to STAT1 in bone marrow-derived macrophages (BMDMs), suggesting that PKM2 may also regulate STAT1 activity 68. Finally, nuclear PKM2 can also modulate STAT5 activity in cancer cells (**Fig. 1**) 69. There are relatively few studies on PKM2 regulating the activation of CD8. PKM2 knockdown in myeloid dendritic cells can reduce the abilities of these cells to promote the activation of CD8^+^ T cells 70. Nevertheless, PKM2 expression predicts poor prognosis of pancreatic cancer, and inversely correlates with intratumoral CD8^+^ T cells 71.

#### Effects of TEPP-46 on the T cell metabolism

TEPP-46 is an allosteric activator of PKM2, which is a small molecule drug that converts PKM2 into a tetramer and inhibits PKM2 nuclear translocation. TEPP-46 seriously affects T cell activation, inhibits the activation and proliferation of Th17- and Th1 cells in vitro and *in vivo*, and simultaneously suppresses T cell-mediated inflammation 72. However, TEPP-46-treated T cells do not affect oxygen consumption rate (OCR) in activated T cells, confirming that TEPP-46 specifically impacts the engagement of glycolysis in T cells 72. Of note, while blocking PKM2 translocation into the nucleus and induction of glycolytic gene, TEPP-46 also increases the activity of PKM2 by inducing its tetramerization 15. This would likely maintain a constant rate of pyruvate flux into the TCA cycle and may be the reason why OCR is not affected by TEPP-46. Importantly, previous work in transformed cells, as well as in immune cells, suggest that, apart from glycolysis, induction of PKM2 tetramerization or knock down of the PKM2 gene may impact engagement of PPP and nucleotide synthesis as well as lipid metabolism 15, 62, 73, 74. TEPP-46 inhibits glycolysis and block mTORC1 activity. Thus, TEPP-46 can also indirectly affect T cell differentiation through HIF-1α/mTOR signaling 61. Taken together, TEPP-46 may have a broader effect on T cell metabolism.

Recent report demonstrated that PKM2 could phosphorylate protein substrates, confirming the potential of such an enzyme to regulate protein functions by post-transcriptional phosphorylation 59. Therefore, taking PKM2 as the pharmacological target, studying new PKM2 activators, or inhibiting the production of PKM2 dimers may represent a valuable therapeutic approach in T cell-mediated cancer immunity.

### PKM2 regulates B cells

Lymphocytes play an important role in the immune response. Generally, B cells require the help of T cells in the immune response. Activation of PKM2 in T cells can regulate the production of B cell antibodies. Extracellular vesicles (EVs) are important as tools in cellular communication. PKM2 activator TEPP46-stimulated T cells produce EVs that promote B cell IgG secretion. In contrast, EVs secreted by PKM2-deficient T cells significantly inhibits B cell mitochondrial programming activation and the production of IgG 75. Therefore, PKM2-mediated EVs in T cells may be an important regulator of B cell production of IgG. Another study showed PKM2 is required to support metabolic reprogramming for homocysteine-induced B cell activation and function 76, so PKM2 is important for immune regulation in the TME.

### PKM2 regulates dendritic cells

In the tumor immune microenvironment, MDSC belongs to a family of bone marrow cells with T cell immunosuppressive function, which can differentiate into DCs, tumor-associated macrophages (TAMs) and granulocytes during the tumor progresses. MDSC express pro-angiogenic factors, inducible carbon monoxide synthase (iNOS), indole 2,3-dioxygenase, etc. It plays a role in promoting angiogenesis, inhibiting innate immunity and adaptive immunity 77, 78. Among these cells, DCs are the important APCs with mature immune activation and regulation, which is expressed as costimulatory molecules (such as CD80 and CD86) 79. DCs activation is a critical step in initiating an immune response through antigen uptake, processing and presentation. The development of DCs requires histone deacetylase (HDAC) 80. Once stimulated by HDAC, the expression of mature markers and cytokines is reduced 81. Trichostatin A (TSA) is a broad-spectrum HDAC inhibitor. TSA induces the expression of DC costimulatory molecules CD80 and CD86, decreases the uptake of FITC-dextran, and promotes DC migration, allowing DCs recruited to hypoxic regions. TSA reduces the pro-inflammatory cytokine IL-1β, IL-10, IL-12 and TGF-β to alter the secretion of cytokines. TSA increases expression of PKM2 gene by up-regulating serine/arginine-rich protein (SRSF3), which is located upstream of PKM2 82. Increasing tumor glycolysis produces more ATP to promote DC migration and antigen presentation. Therefore, the SRSF3-PKM2 pathway affects the activity and function of DCs by TSA through the Warburg effect, and is crucial for cancer cell migration and immune killing.

### PKM2 regulates tumor-associated macrophages

**Figure 2:**
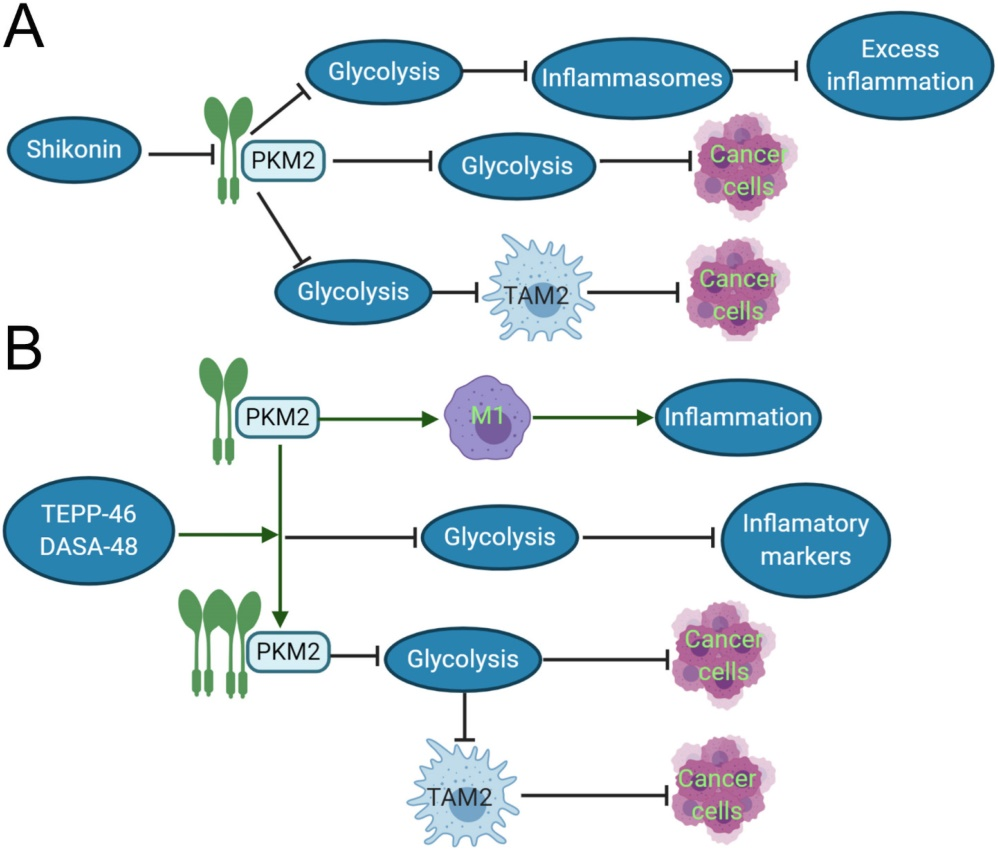
Inhibitor and activator of PKM2 role in the inflammation and cancer development. A. In acute inflammation, shikonin, the inhibitor of PKM2, can reduce the expression of dimeric PKM2, thereby inhibiting glycolysis, reducing the release of inflammasomes, and inhibiting excessive inflammation. In cancer tissues, shikonin can directly affects the Warburg effect and suppress cancer growth by reducing the expression of dimeric PKM2. It also can downregulate dimeric PKM2 to produce lactic acid of aerobic glycolysis, and restraint the polarization of TAM1 to TAM2, so indirectly suppress cancer growth. B. In the inflammatory response induced by LPS, the dimeric PKM2 can promote the transformation of macrophages to M1 type and evoke the occurrence of inflammation. TEPP-48/DASA-48, the activator of PKM2, can convert dimeric PKM2 into tetrameric PKM2, which reduces glycolysis, and then decrease the production of inflammatory markers such as IL-1β, lastly inhibits inflammation. In cancer cells, the PKM2 activator directly reduces the Warburg effect of cancer cells and inhibits caner growth. Otherwise, it can also indirectly suppress cancer growth by reducing the production of lactic acid, which restraint the polarization of TAM2.

TAMs constitute a plastic and heterogeneous cell population of the TME that can account for up to 5% of some solid neoplasms. Most often, TAMs support disease progression and resistance to therapy by providing malignant cells with trophic and nutritional support 83. TAMs execute homeostatic functions and regulate tumor growth, which is highly plastic and can reversibly polarize between the TAM1 and TAM2 phenotypes. These TAMs not only prevent T cells from attacking cancer cells, but also secrete growth factors to nourish cancer cells. They promote cancer angiogenesis and cause cancers to spread through blood vessels. PKM2 can upregulate PKM2-dependent glycolysis by selectively activating the eukaryotic translation initiation factor-2 alpha kinase 2 (EIF2AK2)-dependent NRR family, thereby facilitating the release of IL-1β, IL-18 and high mobility group box 1 (HMBG1) in TAMs 84. The phosphorylation of EIF2AK2, a protein kinase activated by viral infection, has been recently shown to be required for the activation of various inflammasomes in macrophages 85. Lactate promotes EIF2AK2 phosphorylation in macrophages, PKM2 can increase the production of lactic acid through aerobic glycolysis and promote the release of inflammasomes, resulting in excessive inflammation, such as sepsis. Shikonin, a potent PKM2 inhibitor in cancer cells and macrophages 86, can effectively prevent the release of inflammasomes, and findings suggest that pharmacologic inhibition of PKM2 by shikonin selectively suppresses NLRP3 and AIM2 inflammasome activation and reduces sepsis by inhibiting PKM2-dependent glycolysis 84. Shikonin also directly inhibits the growth of cancer cells by suppressing PKM2-mediated Warburg effect 87. The dimeric PKM2 modulates the glycolysis of TAMs and induces the phenotypic transformation of TAM1 to TAM2 (CD163 and CD68 labeled TAM2) 88, which does not play an anti-cancer role in function, but instead participates in various tumor development and invasion 89. Moreover, Shikonin can decrease the expression of dimeric PKM2 and reduce the conversion of macrophages to M2-macrophages 84. The interesting results demonstrated that PKM2 activators such as DASA-58 and TEPP-46 can activate dimeric PKM2 to tetrameric PKM2 90, reduce the transformation of TAM2, and inhibit tumor growth. Both DASA-58 and TEPP-46 also can inhibit LPS-induced glycolytic reprogramming and succinate production 73. Therefore, we can speculate that the use of PKM2 activators and inhibitors with TAM2 as an immunometabolism target combined with traditional treatment can become a new program for the future treatment of cancer patients (**Fig. 2**).

### PKM2 modulates immune checkpoint PD-L1

Under normal conditions, the balance between costimulatory molecules and cosuppressive molecules is upheld and T cell immunity maintains proper depth and breadth. However, cancer cells can abnormally up-regulate co-suppressor molecules and their associated ligands, which are immunological checkpoints. Through the high expression of immunological checkpoint molecules, T cell activation is inhibited, which in turn causes cancer immune escape. Recently, immunological checkpoint inhibitors have attracted attention as one of the most promising immunotherapies, especially the immunological checkpoint PD-L1. PD-L1 is a member of the B7 family of costimulatory/co-suppressor molecules. PD-L1 can express on a variety of cell types including cancer cells. They have shown to have potent immunomodulatory effects through their function as negative regulators of T cells 91. PD-1 is involved in the regulation of T cell activity in peripheral tissues mainly through its interaction with PD-L1 and PD-L2. The discovery of negative regulators of these immune responses is critical for the development of checkpoint inhibitors. This shifts the focus from developing a therapy that activates cancer to the host immune system, to burgeoning a therapy that targets checkpoint inhibitors. When the PD-1 pathway is continuously activated in the TME, T cell function is inhibited and impossible to kill cancer cells. PD-1 or PD-L1 inhibitors, on the other hand, block this pathway and partially restore T cell function, allowing these cells to continue to kill cancer cells. However, studies have shown that about one-third of patients have acquired tolerance after long-term treatment, leading to tumor recurrence. This tolerance may relate with low tumor immunogenicity and hypoxic TME. The therapeutic tolerance mechanism in immune checkpoints could overcome by a new generation of treatment 92, 93. PD-L1 can express in the immune cells (lymphocytes, macrophages) and cancer cells. In particular, the expression of PD-L1 plays an important role in the functional transformation of TAMs.

**Figure 3:**
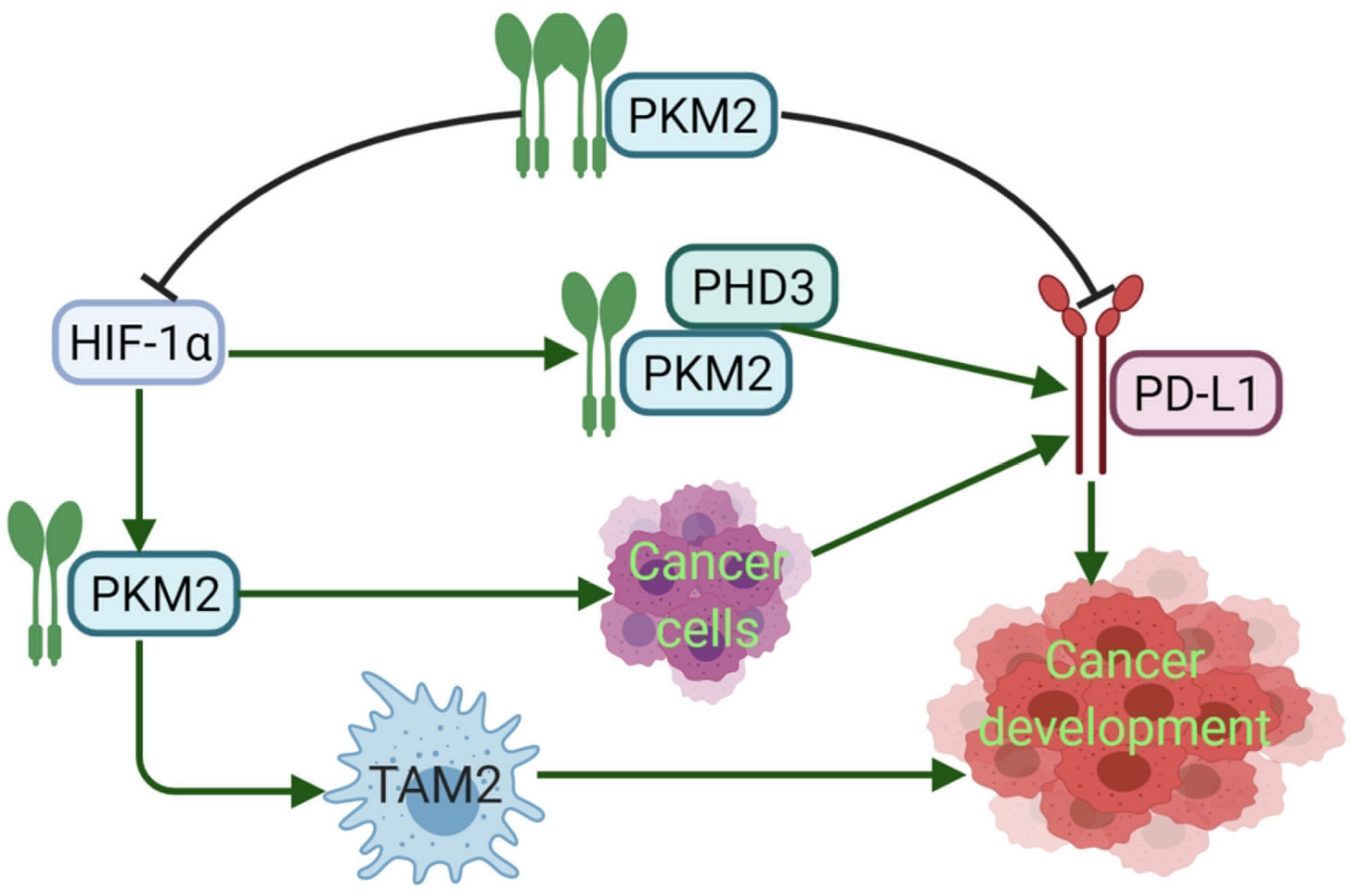
PKM2 regulates the expression of PD-L1. Dimeric PKM2 promotes the transformation of TAMs from TAM1 to TAM2, which is beneficial to the development of cancers. Dimeric PKM2 can also directly promote overexpression of PD-L1 in cancer cells. Dimeric PKM2 acts as a HIF-1α coactivator, which relies on PHD3, to upregulate the expression of PD-L1. However, PKM2 activator such as DASA-58 and TEPP-46, transforms its conformation from dimer to tetramer, inhibits PD-L1 expression and HIF-1α activity, ultimately, suppresses cancer development.

PD-L1 expression requires PKM2. Meanwhile, PKM2 stimulates HIF-1α transactivation, it complements P300 in response to hypoxic conditions and occurs in activated immune cells and cancer cells. During the metabolic reprogramming process, PKM1 expression is decreased and PKM2 expression is increased. PKM2 is mainly present as a monomer or dimer with low enzyme activity. At this stage, PKM2 can be transferred to the nucleus 94. During the complex regulation of HIF-1α and P300, PKM2 acts as a HIF-1α coactivator, which relies on prolyl hydroxylase 3 (PHD3). HIF-1α and PKM2 bind directly to the HRE site of PD-L1, and then promote the HRE subregion and PD-L1 expression. PKM2 is changed from tetramer to dimer, and HIF-1α binds to DNA hypoxia transcripts (HRE), promoting IL-1β transcription and causing inflammatory response. Studies have shown that PKM2 is generally a dimer in cancer cells, which binds to the promoter of PD-L1 in resting mouse bone marrow macrophages resulting in significantly increased PD-L1 expression. However, TEPP-46 can downregulate expression of PD-L1 on macrophages, DCs, and T cells as well as cancer cells in a mouse CT26 cancer model 58. After PKM2 is activated by TEPP-46, PKM2 is converted to a tetramer form, which can inhibit the glycolysis required by cancer cells and HIF-1α activity 73 (**Fig. 3**). Furthermore, in our previous report, PKM2 has significant positive association with PD-L1 expression, and high PKM2 and PD-L1 levels in cancer cells and immune cells can predict the poorest overall survival of patients with lung adenocarcinoma 95, 96. Thus, expression of PKM2 into a tetramer or silencing PKM2 mRNA prevents LPS-induced PD-L1 expression and reduces cancer evasion of immune surveillance.

## Conclusion and future perspective

The malignant progress of cancers is a process of energy consumption, which requires metabolism to provide sufficient energy to maintain basic requires. PKM2 plays a vital role in the immunometabolism by enhancing OXPHOS and the Warburg effect. It not only affects the generation and migration of immune cells, but also supply energy demand for the activation of these cells. Concurrently, PKM2 has an important influence on the immune checkpoint PD-L1. PKM2 builds a bridge between energy metabolism and cancer immunity, if it is hypothesized and established. The activator and inhibitor of PKM2 can both develop to enhance the anti-cancer immune response and inhibit cancer growth and metastasis by regulating the metabolism of cancer cells and immune cells in the TME through HIF-1α/PKM2 pathway. Therefore, a comprehensive understanding of the interaction between PKM2 and immune cells may help to discover new potential targets for cancer therapy.
